# Muscle group dependent responses to stimuli in a grasshopper model for tonic immobility

**DOI:** 10.1242/bio.20135520

**Published:** 2013-09-24

**Authors:** Ashwin Miriyala, Aparna Dutta-Gupta, Joby Joseph

**Affiliations:** 1 Center for Neural and Cognitive Sciences, University of Hyderabad, Hyderabad, AP 500046, India; 2 Department of Animal Sciences, University of Hyderabad, Hyderabad, AP 500046, India

**Keywords:** Habituation, Stimulus response, Tonic immobility

## Abstract

Tonic Immobility (TI) is a prolonged immobile condition exhibited by a variety of animals when exposed to certain stimuli, and is thought to be associated with a specific state of arousal. In our study, we characterize this state by using the reliably inducible TI state of the grasshopper (*Hieroglyphus banian*) and by monitoring abdominal pulsations and body movements in response to visual and auditory stimuli. These pulsations are present during the TI and ‘awake’, standing states, but not in the CO_2_ anesthetized state. In response to the stimuli, animals exhibited a suppression in pulsation and a startle response. The suppression of pulsation lasted longer than the duration of stimulus application. During TI, the suppression of pulsation does not habituate over time, whereas the startle response does. In response to the translating visual stimulus, the pulsations are suppressed at a certain phase independent of the time of stimulus application. Thus, we describe TI in *Hieroglyphus banian* as a state more similar to an ‘awake’ state than to an anesthetized state. During TI, the circuitry to the muscle outputs controlling the abdomen pulsation and the startle response are, at least in some part, different. The central pattern generators that maintain the abdomen pulsation receive inputs from visual and auditory pathways.

## Introduction

Arousal states in organisms, such as the ‘awake’ and sleep-like states, are characterized by differences in motility, responsiveness to stimuli and physiological parameters. The variation in the potential of an animal to respond to an environmental stimulus can be correlated to a characteristic (yet plastic) state of activity of neuronal systems.

Tonic immobility (TI) is considered to be a state of arousal characterized by severe and prolonged immobility. It can be induced in a variety of animals by stressful stimuli, and after an extended duration of immobility, the animal is observed to spontaneously revert back out of the TI state. Within the TI state, it is commonly reported that the animal exhibits a lack of responsiveness to environmental stimuli (Klemm, 1971). TI has been suggested as a behavioral strategy to avoid predation, because predators often rely on detecting movement rather than static features (Bracha, 2004). This is shown to increase survival chances, as was observed in frog tadpoles (Lambert et al., 2004).

TI is shown to be induced in invertebrate models such as beetles (Miyatake et al., 2009), crickets (Nishino and Sakai, 1996) and crabs (O'Brien and Dunlap, 1975) as well as vertebrate models such as lizards, rats, chickens and sharks (Henningsen, 1984). In rats (Meyer et al., 1984) and lizards (Hoagland, 1928), it has been shown that TI can be induced by applying pressure to the sides of the thorax followed by turning the animal dorsum downwards. Bright flashes of light and mechanical stimuli are reported to be enough to invoke TI in potato beetles (Metspalu et al., 2002). In the stick insect and cricket, the femoral chordotonal organ stretch receptors are suggested to play a role in inducing TI (Nishino, 2004; Godden, 1972).

After inducing the behavior, the animal can remain immobile for extended periods of time, and this duration is shown to be dependent on the action of certain hormones (Nishi et al., 2010), neurotransmitters (Hicks et al., 1975) and temperature (Hoagland, 1928). However, little is known about how muscle outputs during this state behave in response to external stimuli. Characterizing these responses is necessary for understanding the TI state.

Here we show that TI can be induced in a grasshopper system and the abdomen pulsations that persist in this state can be used to study the behavioral response to visual and auditory stimuli. We are able to use this paradigm to demonstrate a condition where multiple stimulus modalities can evoke a similar response in the same motor system, while the structure of these responses may differ. Distinct motor systems on the other hand may vary in the nature of their habituation, independent of the stimulus modality.

## Materials and Methods

### Grasshoppers

Adult female grasshoppers *Hieroglyphus banian* hatched and reared in a crowded colony were used in all the experiments. The cage was maintained in a 14/10 hour light/dark cycles at an ambient temperature ranged between 25–30°C.

### Inducing TI

**Fig. 1.**
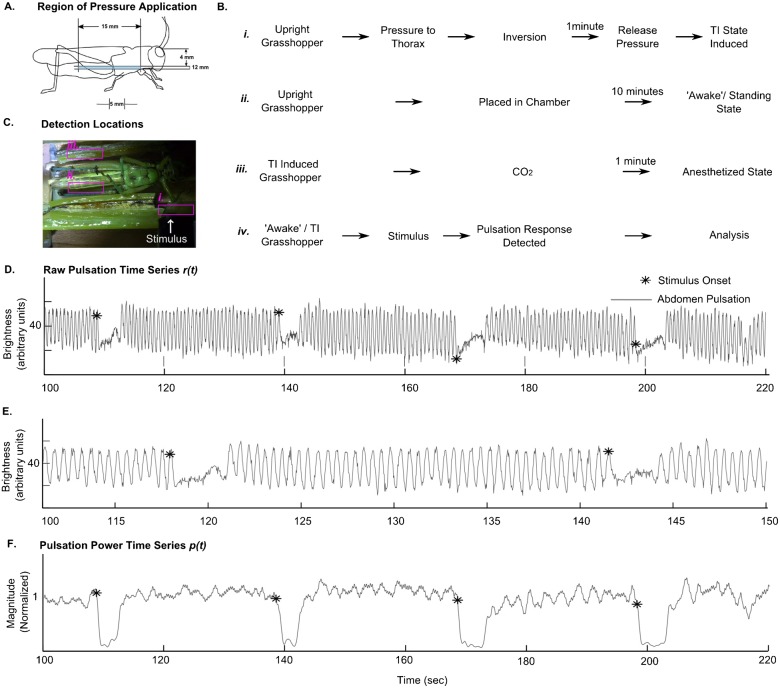
(A) Highlighted region shows where pressure is applied to induce TI in the grasshopper. (B*i*) Procedure for inducing TI. (B*ii*) Procedure for studying ‘awake’, standing state. (B*iii*) Procedure for inducing anesthesia. (B*iv*) Procedure for studying pulsation response to stimuli. (C) Regions in the video used for detecting (C*i*) stimulus onset, (C*ii*) abdominal pulsations and (C*iii*) control region. The brightness of these regions are measured over time to get *r(t)*. (D) Raw abdominal pulsation data during TI. (E) Zoomed *r(t)*. (F) Pulsation strength *p(t)* obtained from smoothened-differentiated-rectified-low pass filtered *r(t)*.

To induce the TI state, the grasshopper was placed between two clamps on its back in such a way that it was held by the sides of the thorax just below the forelegs and midlegs (Fig. 1A). The animal is kept in this position for 1 minute, after which the clamps are released. At this point, the grasshopper is lying on its back parallel to the ground with its tarsi not having contact with any surface, and it is free to move and upright itself. The TI state is considered to be induced if the animal remains motionless after the clamps are released (Fig. 1B shows the outline of this procedure).

This immobility lasts for a varying duration of the order of minutes, and the abdominal pulsations are quantified during this time. For analysis purposes, only those animals that remained immobile for at least 300 seconds were considered. For each animal, at least 25 minutes were given between subsequent inductions of TI, and all the tests were conducted during daylight hours, in a well-lit closed environment.

### Video recording the TI and ‘awake’ states

The video-camera (Sony Handicam HDR-XR500, Japan) was placed such that the entire grasshopper was in its field of view, and the abdominal pulsations were clearly visible. The focus, exposure and white-balance were manually adjusted to keep them constant throughout the video. The video file format is SD 9M (HQ), the resolution is 720×480 and the frame rate is 29.97 fps. The sound track in the video recordings has a sampling frequency of 48 kHz.

To record animals in the TI state, the recording was started from the moment the clamps were pulled apart, and ended when the grasshopper spontaneously woke up, or at the 15 minute mark, whichever came first. For the standing, ‘awake’ state recordings, the animal was kept in a wooden chamber with a transparent bottom to allow for recording. The dimension of the chamber was such that movement was allowed, and thus only the lengths of the recordings where the abdomen pulsations could be tracked were used.

### Comparing movement between TI and ‘awake’ states

The video file was processed using MatLab. To compare the TI and standing states, body movements were monitored in both states. To detect only gross movements, first the difference between the n^th^ frame and (*n*+3)^rd^ frame was computed. From this time series of frames the means of regions around the midlegs, forlegs and mouth were calculated for each frame. The resultant time series were detrended and normalized by their respective root mean square values, resulting in a time series *m(t)* for each detection region (supplementary material Fig. S1). Since regions near the head of the animal get blocked every time the visual stimulus is given, and due to the presence of the startle response, a 3 second window after each onset of the visual stimulus in *m(t)* was replaced with its baseline value. Movement analysis was done on the same data set as for other analysis.

The time points of movement were found for both the ‘awake’ animals (*n* = 5 animals) and TI animals (*n* = 13 animals) as those points where *m(t)* was greater than 5 units (supplementary material Fig. S1). In the case of the ‘awake’ animals, the movement did not affect the abdomen pulsation analysis since the movements were only that of midleg, foreleg and mouth parts.

### Quantifying the abdominal pulsation in TI, anesthetized and ‘awake’ states

We observed that the abdominal pulsations were associated with a change in brightness of the abdominal segments, and therefore we used the brightness as a surrogate measure to quantify the pulsations. For all three states, the brightness was estimated as the mean of the pixel values in a region of the abdomen at each time point of the video. The same was done for a control region outside the region of pulsation. A length of the abdomen between the 1st and 4th segments was chosen for detection since it shows most prominent changes in brightness with phases of ventilation (Fig. 1C*i*).

The resulting raw time series is labeled as *r(t)* (Fig. 1D,E). *r(t)* was smoothened to remove high frequency noise and then differentiated and detrended to get the rhythmic component and remove DC components. This new time series was full-wave rectified and low pass filtered with a zero-lag triangular filter of length 29. The resultant time series *p(t)* shows a larger magnitude when pulsations are present and a magnitude close to 0 when the pulsation is suppressed (Fig. 1F). This magnitude of pulsation is used as the measure of the pulsation strength. The time series of a control region (Fig. 1C*ii*) undergoes the same filtering process.

Anesthesia was induced by directing a CO_2_ stream in the posterior-anterior direction to a grasshopper in the TI state (Fig. 1B*iii*). A detrended *r(t)* time series obtained as described above was used to compare the power spectra of two 12 second windows, one before CO_2_ application and one 60 seconds after, by using a fast Fourier transform. The grasshopper was confirmed to be in the anesthetic state by observing the recovery in each case.

### Stimulus application and detection

**Fig. 2.**
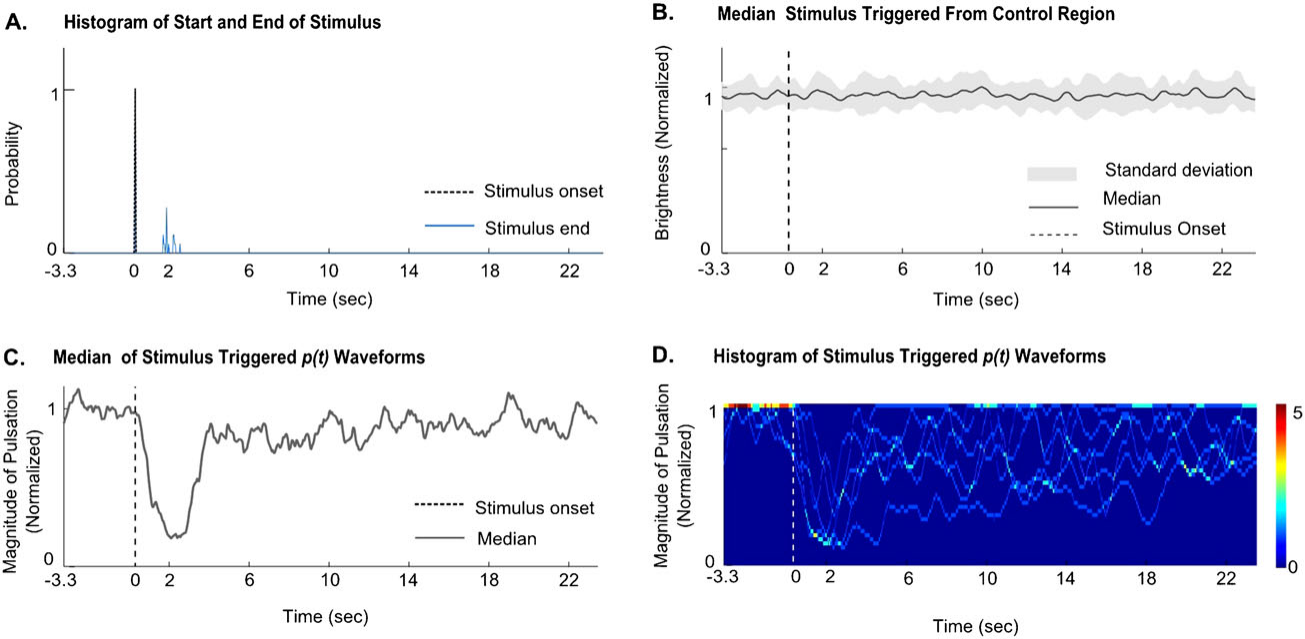
Example of analysis of data for one animal. (A) Probability of start and end of the stimulus triggered around the stimulus onset. (B) Brightness measure in the control region shows no response to stimuli. (C) The median of stimulus triggered *p(t)* waveforms over 6 trials for one animal shows a decrease in pulsation magnitude in response to a translating visual stimulus (*n* = 1 animal, 6 trials). (D) Histogram of pulsation data at each time point.

The visual stimulus is a black rectangular physical object that subtends an angle of 80 degrees at the eye of the grasshopper. It is remotely and manually operated, and is set to traverse laterally in the visual field of the grasshopper at a height of approximately 30 mm above its eye level. Starting outside the animal's visual field, the stimulus travels an angle of 80 degrees in its visual field, and is then retracted along the same path. The entire stimulus presentation lasts approximately 2 seconds, and was repeated every 30 seconds. The mean brightness of the visual stimulus detection region is calculated per frame (Fig. 1C*iii*), and the time series obtained from this region is smoothened, differentiated and thresholded to determine the time point of onset and end of the stimuli (Fig. 2A).

**Fig. 3.**
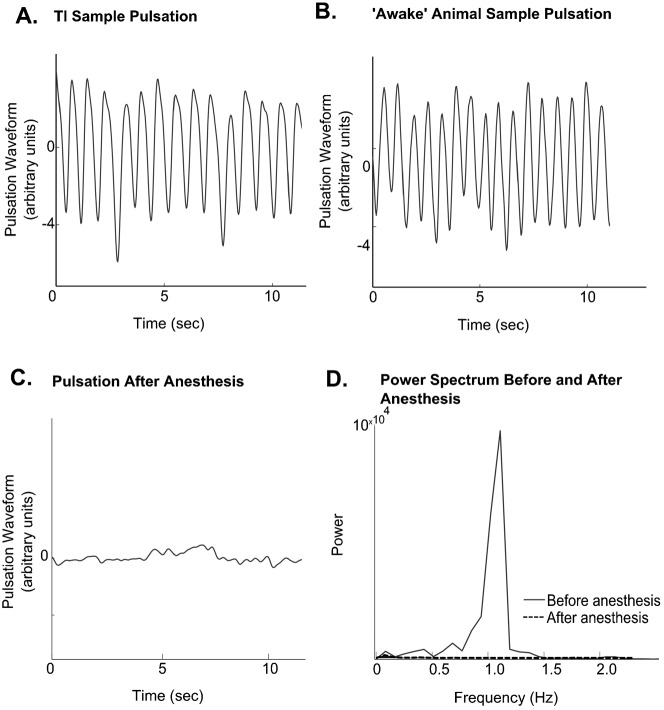
(A) Smoothened *r(t)* during TI. (B) Smoothened *r(t)* during the ‘awake’ state. (C) Pulsations stop after CO_2_ anesthesia, confirmed by comparing the power spectrum before and during anesthesia (D).

The sound stimulus was a clapping sound administered via speakers which were directed away from the grasshopper's body and measured 75.9±0.7 dB (mean ± std) at the grasshopper's location. It was applied once every 30 seconds. The sound is recorded along with the video through the microphone on the camera (sound spectrogram and power spectral density shown in supplementary material Fig. S2A,B). This time series was extracted from the video file and was first rectified, and then filtered using a second order Butterworth low pass filter with cut-off at 3 Hz, to estimate the power at any given time. This time series is then thresholded to detect the onset of the sound stimuli.

### Constructing stimulus triggered time series

Waveforms were constructed from *p(t)* for each of the first 6 stimuli, starting 3.3 seconds before each stimulus. Each of these stimulus triggered *p(t)* waveforms was normalized with respect to the mean of its first 3.3 seconds of time series data, which corresponds to the baseline abdomen pulsation strength. This allows for a comparative analysis to be made between the time series of the pulsation strengths. The median of these stimulus triggered *p(t)* waveforms for one animal is plotted in Fig. 2C. Median was used because the distribution of responses was not Gaussian; pulsations were either present or were suppressed.

### Constructing response triggered time series

We wanted to check whether the decrease in magnitude of the abdomen pulsation in response to the visual stimulus would occur at a specific phase of the pulsation cycle, independent of when the stimulus was presented. We also wanted to observe the nature of the pulsations before and after recovery from the suppression of pulsation. Since the pulsation frequency differs between animals and is observed to change over time, the frequencies of each of the stimulus triggered *r(t)* waveforms were resampled to have the same frequency (chosen as the mean of the original frequencies). This allows for a comparison of the structure of the waveform before and after the deviation of the sinusoidal pattern. The resampled stimulus triggered *r(t)* time series were detrended and smoothened to get the frequency normalized waveform, *s(t)*. Supplementary material Fig. S4Ai shows the stimulus triggered *r(t)* trials of two different animals, while supplementary material Fig. S4Aii shows the corresponding *s(t)*.

As the pulsation is periodic, the magnitude of deviation from the sinusoid is a measure of onset of the response. The deviation time point is found by differentiating *s(t)*, and then scaling it to get the cosine of *s(t)*, which is then 90 degree phase shifted to get *ŝ(t)*. *s(t)* is the estimate of *s(t)*, if the original time series was purely sinusoidal. *d(t) = |s(t)−ŝ(t)|* was then computed; a large value in *d(t)* occurs where the waveform changes from being sinusoidal in response to the stimulus. The differential of *d(t)* was thresholded to find the time point of onset of the response. A window around this point was taken from *s(t)* to get the response onset triggered pulsation waveform. Such a response onset triggered *s(t)* waveform was obtained for each trial (average of response onset triggered *s(t)* waveforms shown in Fig. 7A; supplementary material Fig. S4B shows examples of *s(t)*, *ŝ(t)* and *d(t)* waveforms).

If the response is locked to a particular phase of pulsation, triggering from the response should cause the sinusoids to add up in phase before the response onset. Thus the amplitude *A_m_* of the cycle immediately preceding response onset (Fig. 7A) should be greater than the maximum amplitudes obtained from averages of randomly triggered *s(t)* waveforms. We tested this by finding the probability of getting such maximum amplitudes to be *A_m_* or greater (Fig. 7B).

### Measure of startle response

After inducing TI, only animals that showed a startle to at least one visual or sound stimulus trial were taken for measuring the startle response. Startle detection was done the same way as the body movement detection as described above, by monitoring a region encompassing the initial and final positions of the left hind limb tarsus, resulting in movement time series *m(t)*. The maximum amplitude change, which corresponds to the startle response, occurred 0.4–0.7 seconds after the visual stimulus onset, and 0.0–0.3 seconds after the sound stimulus onset. Therefore after generating stimulus triggered *m(t)* waveforms for the first 6 trials for each animal, the means of these regions were compared using a two-way ANOVA. The detection regions for the two stimuli differ since the sound stimulus was observed to elicit a faster startle response than the visual stimulus (supplementary material Fig. S2C).

### Statistical analysis

To test whether the magnitude of pulsation significantly decreased in response to the stimuli, a response period (1.2 to 2.2 sec after visual stimulus onset, and 0.2 to 1.2 sec after sound stimulus onset; refer to Fig. 5A) of the median of stimulus triggered *p(t)* waveforms was compared to a pre-stimulus period (−3.3 to −2.3 sec, with reference to the stimulus onset) and a post-response period (20 to 21 sec).

To test for habituation, the response periods of the medians of stimulus triggered *p(t)* waveforms of each of the first 6 trials were compared. Comparisons were made using the Kruskal-Wallis test followed by a Tukey-Kramer post hoc test.

The durations of immobility were obtained from the median of stimulus triggered *p(t)* waveforms, using the full width at half maximum parameter. This parameter gives the distance, in seconds, between points on the waveform where the magnitude reaches half its lowest value.

### Recording abdominal neuro-muscular activity using copper electrode

The field potentials due to the neuro-muscular activity were recorded to check the coherence with the abdominal pulsation movement detected by the video recording. Recordings were taken from the 190–194 abdominal muscle groups (as described in *Schistocerca gregaria* by Lewis et al., 1973) using an insulated copper wire of diameter 0.3 mm, with insulation removed at the tip. The wire was inserted through an incision made at the base of the third abdominal segment, and was held to the cuticle using candle wax. It was then attached to the animals back with wax before connecting it to the head-stage amplifier (Axon Instruments CV203BU Head-stage). A silver-silver chloride ground wire was inserted approximately 5 mm into the thorax. The head-stage amplifier was connected to the main-stage amplifier (Axoclamp 200 B, Molecular Devices, Union City, CA, USA) set to a gain of 5×, through a digitizer (NI USB 6211), and LabVIEW was used to record the data (sampling rate 5000). The animal was kept upright in a small chamber kept in a Faraday cage, and its abdominal pulsations were video recorded.

**Fig. 4.**
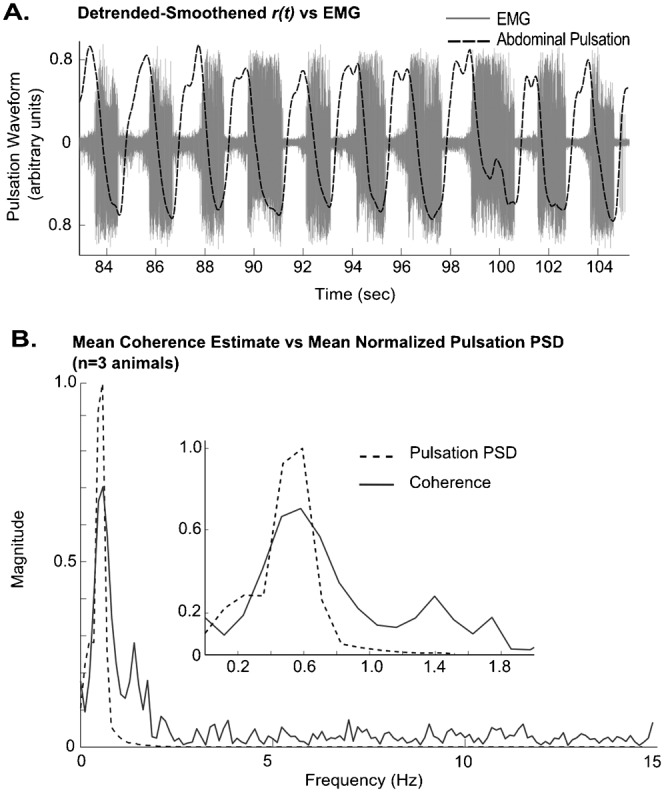
(A) An example trace of the high frequency band-pass filtered neuro-muscular field potential recording (solid trace) and the smoothened *r(t)* (dashed trace). (B) Mean coherence estimate between pulsation and field potential, showing a coherence of 0.7 at 0.5 Hz (solid trace). The dashed trace shows the peak normalized mean power spectral density (PSD) of the abdominal pulsations (*n* = 3). Inset shows zoomed region of maximum coherence and maximum frequency.

The field potential data was first filtered using a 2nd order high frequency band-pass filter at 200–1000 Hz, and was then rectified and smoothened to get its power estimate (sample band-pass filtered trace shown in Fig. 4A). Both the smoothened *r(t)* from the video and the field potential power series data were then resampled to 30 Hz, and the magnitude squared coherence between the two detrended time series vectors was measured using Welch's averaged periodogram method, with a Hanning window of 256 points, 128 point overlap and 256 point fft.

## Results

TI could be induced and maintained in 77% of the attempts (successfully induced TI in 34/44 attempts). The median duration of immobility was 540;245 seconds (median; iqr; *n* = 38 animals). In our experiments, the observations were curtailed at 540 seconds, after which the animal would upright themselves on touch. Animals did show immobility durations up to 30 minutes in our setup, but we are not sure how much longer than 30 min that the state could last as we terminated the observations after this time.

### TI is quantifiably different from the ‘awake’ state

To observe and compare TI to the standing, ‘awake’ state, the animal was placed in a chamber and the abdominal pulsations were recorded when gross movements stopped. Even in this condition the animal exhibits movements in its forelegs, midlegs and palps. After quantifying the movement in the ‘awake’ animals, the time points of movements occurring above a threshold were detected for each of these body parts (see Materials and Methods; supplementary material Fig. S1), and were compared with the movements detected in the TI state using the same threshold. It was found that 100% of the ‘awake’ animals showed one or more movements (*n* = 5 animals) in a 295 second time period, whereas none of the TI animals showed any movement in the same time period (*n* = 13 animals).

### Abdomen pulsation persists in TI but not in anesthetized state

There is a regular pulsation (contraction and expansion along dorso-ventral axis, see supplementary material Movie 1) during TI as well as in the upright awake condition. The pulsation data shows a uniform pulsation frequency at 1.36±0.08 Hz (mean ± ste) at the beginning of TI, and this decreases to 0.92±0.10 Hz (mean ± ste; t-test, *P*<0.001, *n* = 14 animals) 240 seconds later. Similarly, for a standing grasshopper the pulsations are readily detectable, having an initial pulsation frequency of 1.09±0.12 Hz (mean ± ste). This frequency decreases significantly by 240 seconds to 0.78±0.09 Hz (mean ± ste; t-test, *P*<0.05, *n* = 14 animals). Fig. 3A,B show sample traces of the raw pulsation data *(r(t))* during the TI state and ‘awake’ state.

We next wanted to observe the nature of these abdominal pulsations in an anesthetized grasshopper. On applying CO_2_ to a grasshopper in the state of TI, we saw that there were no abdominal pulsations (*n* = 3 animals; Fig. 3C,D). On removing the CO_2_, all animals recovered from anesthesia within 10 minutes.

### Visual stimulus suppresses abdominal pulsation in TI and ‘awake’ states

**Fig. 5.**
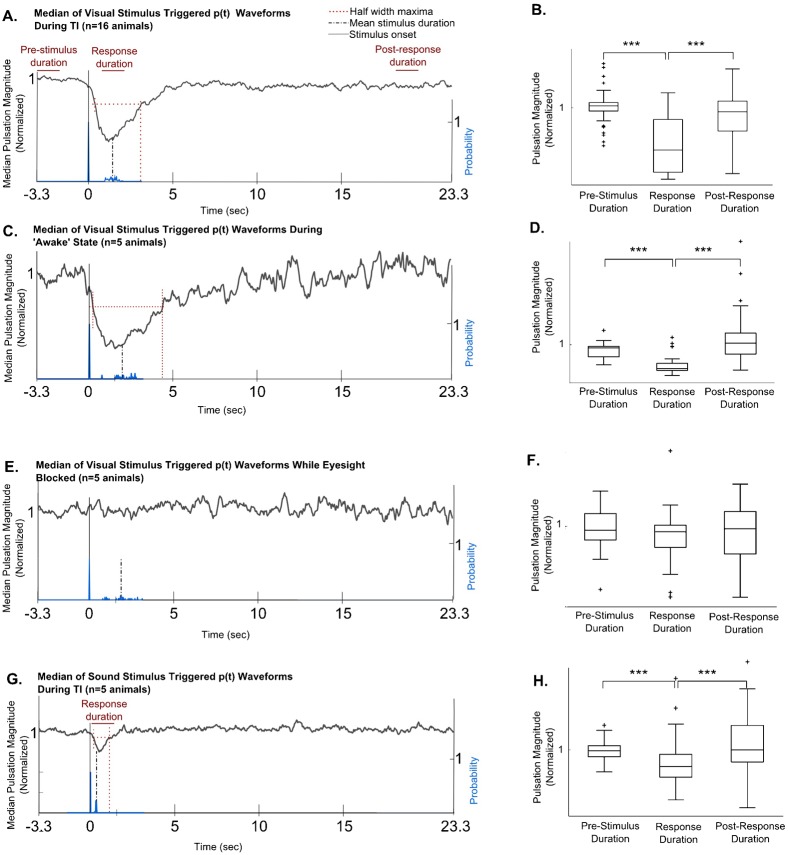
(A,B) The magnitude of pulsation is significantly reduced in a TI induced animal in response to a visual stimulus (*P*<0.001 by Kruskal-Wallis test for medians; *n* = 16 animals). (C,D) The magnitude of pulsation is significantly reduced in an ‘awake’ animal in response to a visual stimulus (*P*<0.001 by Kruskal-Wallis test for medians; *n* = 5 animals). (E,F) After blocking the visual field of the grasshopper, there was no suppression response (*P*>0.3 by Kruskal-Wallis test of medians; *n* = 5 animals). (G,H) The magnitude of pulsation is significantly reduced in response to a sound stimulus when the animal is in the TI state (*P*<0.001 by Kruskal-Wallis test of medians; *n* = 5 animals). In all cases, the full width at half maximum indicates that the response duration lasts longer than the mean stimulus application duration.

During TI, the magnitude of pulsation significantly decreased in response to a translating visual stimulus (*P*<0.001 by Kruskal-Wallis test for medians; *n* = 16 animals, 6 trials per animal; see Materials and Methods). This suppression lasts for a duration (3.33±0.04 sec; mean ± ste) which is longer than the stimulus application duration (1.05±0.03 sec; mean ± ste), as indicated by the full width at half maxima parameter (Fig. 5A,B).

A similar behavior was exhibited by the ‘awake’ animal. The magnitude of pulsation significantly decreases in response to the translating visual stimulus (*P*<0.001 by Kruskal-Wallis test for medians, *n* = 5 animals, 6 trials per animal). The full width at half maxima parameter shows that the duration of the response (4.71±0.07 sec; mean ± ste) lasts longer than the mean stimulus duration (2.16±0.12 sec; mean ± ste; Fig. 5C,D). A control region shows no such response (supplementary material Fig. S3Ai,ii).

To confirm that the response to translational visual stimuli was not caused by any mechanical components originating from the stimulus application, the grasshopper's line of sight of the visual stimulus was blocked. This treatment eliminated the decrease in magnitude of pulsation (*P*>0.3 by Kruskal-Wallis test for medians; *n* = 5 animals, 6 trials per animal), showing that the behavioral response was due to translating visual component of the stimulus (Fig. 5E,F).

### Sound stimulus decreases the magnitude of abdominal pulsation during TI

To test if another stimulus modality can decrease the magnitude of pulsation we tested the response to a sound stimulus during the TI condition. The stimulus caused a significant decrease in the magnitude of pulsation (*P*<0.001 by Kruskal-Wallis test for medians, *n* = 9 animals, 6 trials per animal). This response duration lasts longer than that of the stimulus application duration (0.36 sec), as shown by the full width at half maxima parameter (1.2±0.04 sec; mean ± ste; Fig. 5G,H). The response was faster and of shorter duration than the response to the visual stimulus. A control region shows no such response (supplementary material Fig. S3A*iii*).

### Translating visual stimuli stop the rhythmic pulsation at a particular phase of a cycle

To observe the nature of the response in the abdominal pulsation when the stimulus is applied, all the visual stimulus triggered *r(t)* waveforms were first resampled to one frequency value to get *s(t)*, and then the response onset point was detected (see Materials and Methods). The average of response onset triggered *s(t)* waveforms makes it apparent that the response onset occurs at a specific phase in a cycle, irrespective of when the stimulus was applied (Fig. 7A; *n* = 14 animals, 6 trials per animal). This was tested by comparing the amplitude *A_m_* of the pulsation cycle immediately prior to the response onset with the maximum amplitude of cycles from averages of randomly triggered *s(t)* waveforms. From this we could calculate that the probability of getting an amplitude greater than or equal to *A_m_* was 0.0001 (*n* = 3000 randomly triggered averages; Fig. 7B).

The amplitude of the cycle before the stimulus onset in the average of stimulus triggered *s(t)* waveforms is not significantly different from the maximum amplitudes obtained from averages of randomly triggered *s(t)* waveforms, indicating that the stimulus was not applied at a particular phase in the pulsation cycle (Fig. 7C).

### Abdomen pulsation is coherent with the activity of abdominal ventilatory muscle groups 190–194

To test whether the pulsation movements detected by the camera were coherent with the activity of the abdominal muscles, neuro-muscular field potential recordings were taken from the 190–194 abdominal muscle groups (as described in *Schistocerca gregaria* by Lewis et al., 1973). These muscles are reportedly ventilatory muscles responsible for the dorso-ventral abdominal pumping movements which we monitor in the experiments. A coherence measure between the field potential and pulsation indicated a coherence of 0.7 at 0.5 Hz (*n* = 3 animals; Fig. 4B).

### Magnitude of pulsation response to stimuli during TI does not habituate, whereas startle response does

**Fig. 6.**
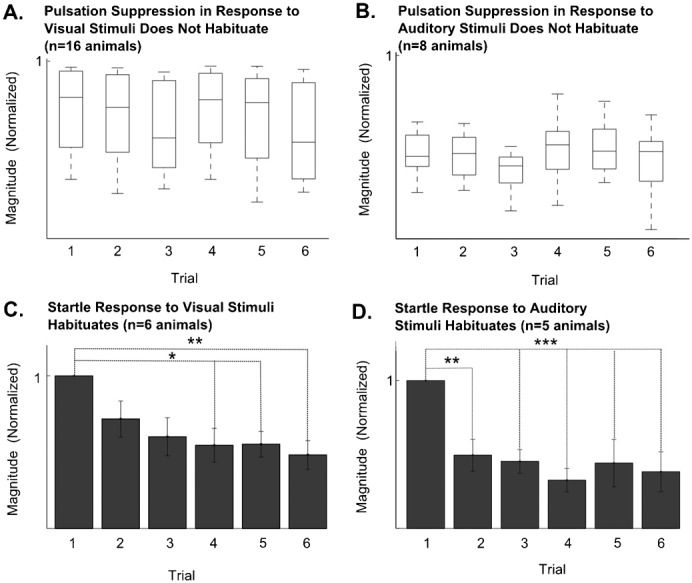
(A) There is no habituation in the magnitude of pulsation suppression in response to visual stimuli over the first 6 stimulus applications (two-way ANOVA, *P*>0.3; *n* = 16 animals). (B) No habituation is observed in response to auditory stimuli either (two-way ANOVA, *P*>0.3; *n* = 8 animals). (C) The startle response to visual stimuli, however, habituate over the first 6 trials (two-way ANOVA, *P*<0.01; *n* = 6 animals). (D) A habituation in response to the auditory stimuli is also observed over the first 6 trials (two-way ANOVA, *P*<0.001; *n* = 5 animals).

It is often observed that the response of an organism to repeated stimuli habituates. To test whether habituation occurs in response to the visual and auditory stimuli during TI, the magnitude of response to the stimuli were compared over the first 6 stimulus applications. No significant decrease was observed in the magnitude of pulsation, to both visual (*P*>0.3 by Kruskal-Wallis test for medians; *n* = 16 animals) and sound (*P*>0.3 by Kruskal-Wallis test for medians; *n* = 14 animals) stimuli. This indicates that the magnitude of pulsation response does not habituate over the first 6 stimulus applications (Fig. 6A,B).

**Fig. 7.**
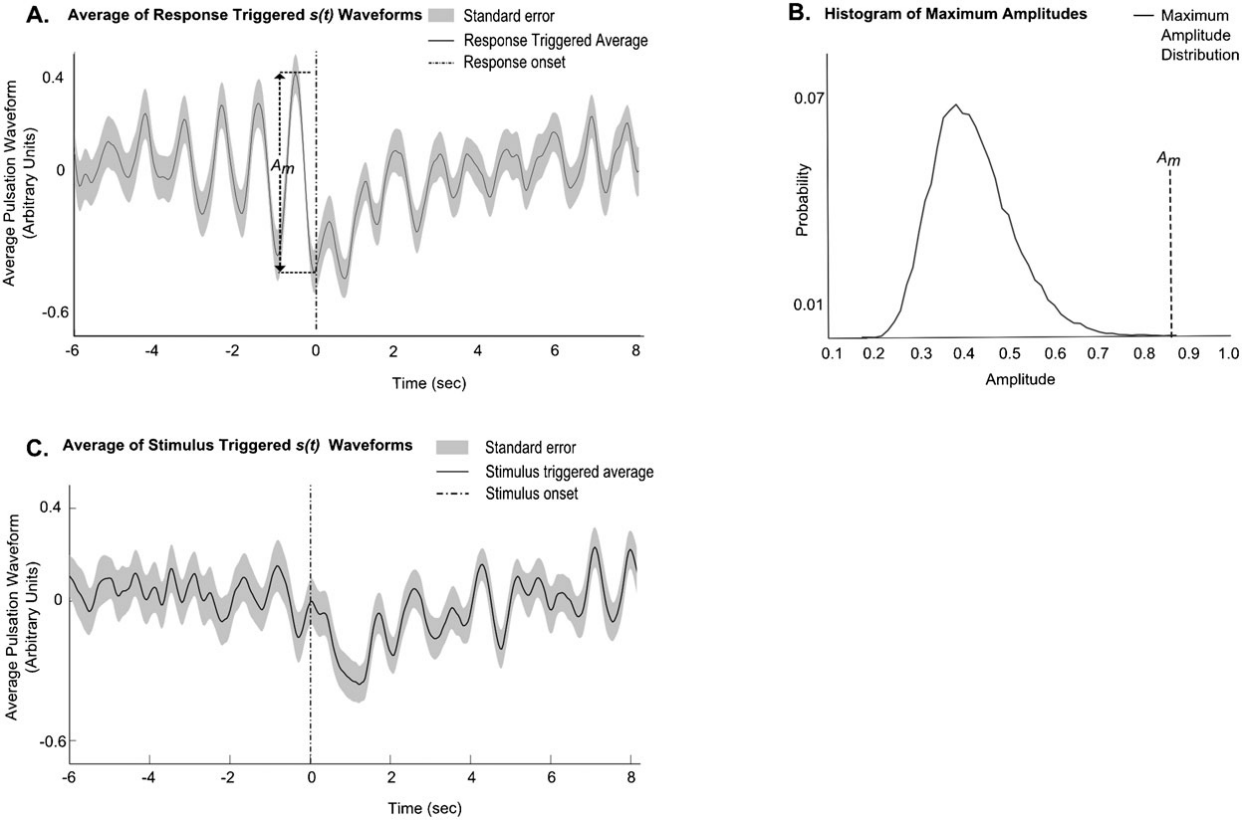
(A) Average of response onset triggered *s(t)* waveforms makes it apparent that the pulsation stops at a specific phase in the cycle. *A_m_* is the amplitude of the cycle before the onset of the response (*n* = 14 animals, 6 trials per animal). (B) Histogram of amplitudes from averages of randomly triggered *s(t)* waveforms (*n* = 3000). The probability of getting an amplitude greater than or equal to *A_m_* is 0.0001. (C) Triggering around the stimulus shows that the stimulus itself was not applied at any specific phase of the pulsation cycle.

It was observed that some animals show an abrupt flick of the hind limbs in response to both translating visual stimuli and auditory stimuli. This startle response was quantified as described in Materials and Methods. Unlike in the case of the pulsation response, the startle response was observed to habituate over 6 stimulus trials, for both stimulus modalities. In response to a visual stimulus the startle response to the 6th trial is significantly less than the startle response to the first stimulus (two-way ANOVA, *P*<0.01, *n* = 6 animals). In response to the sound stimulus, the 3rd to 6th responses were shown to be significantly less than the first (two-way ANOVA, *P*<0.001 *n* = 5 animals; Fig. 6C,D). There is no habituation in the control region (supplementary material Fig. S3B*i*,*ii*).

## Discussion

We were able to induce the TI behavior in a grasshopper model in 77% of the attempts. The median duration of immobility was 540;245 seconds (median; iqr; *n* = 38 animals). This duration is in the same range as that observed in other insects (Nishino and Sakai, 1996; Metspalu et al., 2002; Miyatake et al., 2009).

We show that the TI state is associated with a suppression of body movements other than the abdomen, as indicated by no detected movement in the midlegs, forlegs or palps, whereas all of the standing, ‘awake’ grasshoppers exhibited movement in these body parts. This clearly distinguishes the TI state from the ‘awake’ state. As far as we know, this is the only parameter that distinguishes these states behaviorally. Existing work has addressed certain mechanical and physiological properties within the TI and ‘awake’ states. It has been shown that during tonic immobility in the cricket *Gryllus bimaculatus*, in the metathoracic flexor muscle, slow-excitatory units show persistent firing and common inhibitory motor neurons are suppressed. However in the quiescent state (standing period of no movement), the firing rate of the slow-excitatory units remained less and the common inhibitory motor neurons fired sporadically (Nishino, 2004). Quiescence is also reported to be associated with a higher respiration rate, lower heart rate and shorter durations of immobility as compared to the TI state (Nishino and Sakai, 1996).

The abdominal pulsations were used as a quantification parameter because they are readily detectible. In both the TI and ‘awake’ states, the pulsations are initially uniform, and decrease in frequency over a 240 second period. The magnitude of pulsation decreases in response to a translating visual stimulus, and this response duration lasts longer than the duration of stimulus application. This observation is unlike what is reported in a beetle system, where there is no change in abdominal pulsation in response to a flash of light or mechanical stimulation (Metspalu et al., 2002). However, in the anesthetic state (induced by CO_2_) the pulsations are absent. Thus the presence of abdominal pulsations can be used to describe TI as a state similar to the ‘awake’ state and distinguish it from the anesthetized state.

Similar to the translating visual stimuli, the magnitude of pulsations are also decreased in response to sound stimuli, with the response duration lasting longer than the stimulus application duration, although the response is initiated earlier and is of shorter duration than the response to the visual stimulus. The nature of the response is also shown to differ between the two stimulus modalities. In response to the translating visual stimulus, the pulsation stops at a specific phase of the ventilatory cycle irrespective of the phase in the pulsation at which the stimulus is applied. This suggests the presence of a central pattern generator, which cannot be interrupted by translating visual stimuli in its ongoing cycle other than at a particular phase.

A similar analysis of the pulsation waveform in response to sound stimulus could not accurately identify the point of deviation from baseline pulsation, partly because the response was not as prominent. However, there seems to be a marked decrease in variance which occurs in the downwards slope after the pulsation response to the sound stimulus (supplementary material Fig. S5).

The detected pulsations are controlled by the 190–194 groups of abdominal dorso-ventral expiratory/inspiratory muscles (as described in *Schistocerca gregaria* by Lewis et al., 1973), as confirmed by its coherence with the neuro-muscular field potential recordings. In *Schistocerca gregaria*, the control of the ventilation phases is described by the presence of two coordinating interneurons which run from the metathoracic ganglion to the last abdominal ganglion, which are responsible for initiating and maintaining the duration and intensity of the expiratory motor bursts within each segment. A burst forming pacemaker present in the metathoracic ganglion is suggested to receive input from command fibers which respond to CO_2_, oxygen and other factors, and inhibit the coordinating interneurons during inspiration.

The lobula giant movement detector (LGMD)/descending contra-lateral movement detector (DCMD) system of interneurons are known to show activity in response to translating visual stimuli and thus may play a role in the suppression of the abdominal pulsation via the DCMD. The DCMDs are directly postsynaptic to the LGMD, which is a neuron in the optic lobe that receives input from the entire visual hemifield. In *Schistocerca americana*, the DCMD has been shown to synapse onto motoneurons responsible for evoking escape behaviors (Fotowat et al., 2011), although its effect on other muscle groups is not known. In *Locusta migratoria*, pathways carrying signals from the DCMD as well as acoustic signals are both shown to synapse onto interneuron 724, which possibly indicates a similar pathway of action for the sound stimulus (Baader, 1991).

In some animals in TI, there we observed a startle response in response to the visual or auditory stimuli, characterized by a small twitch in the hind limbs. This twitch could be mediated by same substrate responsible for the jump or kick initiation as described in *Locusta migratoria*, which involves a femoro-tibial flexion brought about by co-activation of hindleg flexor and extensor tibiae motoneurons. It is known that spontaneously or in response to a stimulus, the kick occurs when the extensor generates enough isometric force (Pearson and O’Shea, 1984). In TI, the startle response was observed to habituate over successive applications of visual and sound stimuli. However, the magnitude of pulsation suppression did not habituate to either stimulus. This demonstrates a case where habituation occurs based on the neuro-musculature involved, rather than the stimulus modality.

To further characterize the pathways that are active during TI, responses to odor stimuli should also be looked into. In pigeons, suppressing olfaction is shown to increase durations of TI (Dornfeldt and Bilo, 1990). The effect of looming stimuli should also be tested, as the response to these stimuli in *Locusta migratoria* are shown to habituate (Gray, 2005). Also, in *Schistocerca americana*, the LGMD/DCMD system of interneurons are known to participate in evoking an escape response preferentially to looming visual stimuli (Peron and Gabbiani, 2009).

If the animal is dropped after inverting and applying pressure, to mimic a predator dropping the animal, TI is not observed to be maintained. Thus we are not sure whether TI can be considered as a mechanism to avoid predation. Also, the visual stimulus itself does not seem to play a role in how long the behavior lasts, since the time taken for the animal to wake up after the last stimulus was applied varied between 2 seconds to 30 minutes. This is not in favor of a strategy where the animal is waiting for an opportunity to escape. Although we are not able to conclude the ecological significance on these lines, we still are able to describe a unique behavioral state and describe its characteristic responses to external stimuli.

Overall, our work describes TI as a state similar to the standing ‘awake’ state on the basis of presence of uniform abdominal pulsations and a significant decrease in the magnitude of pulsation in response to visual stimuli. Yet TI differs from the ‘awake’ state since it does not exhibit any non-abdominal spontaneous body movements. We also distinguish TI from the anesthetic state based on the presence or absence of abdominal pulsations. A visual stimulus is shown to decrease the magnitude of the pulsations starting at a particular phase in the pulsation cycle, which suggests the presence of a central pattern generator that receives information from the visual pathway and influences the abdominal muscles. This abdominal muscular output does not habituate to visual or auditory stimuli. However, the muscular output responsible for the startle response does habituate, independent of the stimulus modality.

## Funding

J.J. was supported by the DST-Ramanujan Fellowship. J.J. and A.D.-G. were supported by the grant under UPE2 at the University of Hyderabad.

## Supplementary Material

Supplementary Material
