# Supplementary material for: Muscle group dependent responses to stimuli in a grasshopper model for tonic immobility
Source: Biol Open. 2013 Sep 24;2(11):1214–22. doi: 10.1242/bio.20135520 (PMC3828768; doi:10.1242/bio.20135520)
Supplement: Supplementary Material [file supp_2_11_1214__index.html]

Muscle group dependent responses to stimuli in a grasshopper model for tonic immobility — Muscle group dependent responses to stimuli in a grasshopper model for tonic immobility — Supplementary Material 

# Muscle group dependent responses to stimuli in a grasshopper model for tonic immobility

## bio.20135520 Supplementary Material

**Files in this Data Supplement:**

- Supplementary Material - Ashwin Miriyala et al. doi: 10.1242/bio.20135520
- Movie 1 - **Movie 1. Response to translating visual stimuli in tonic immobile state.**
