# Supplementary material for: Muscle group dependent responses to stimuli in a grasshopper model for tonic immobility
Source: Biol Open. 2013 Sep 24;2(11):1214–22. doi: 10.1242/bio.20135520 (PMC3828768; doi:10.1242/bio.20135520)
Supplement: Supplementary Material [file supp_bio.20135520_bio.20135520-s1.pdf]

Supplementary Material

Ashwin Miriyala et al. doi: 10.1242/bio.20135520

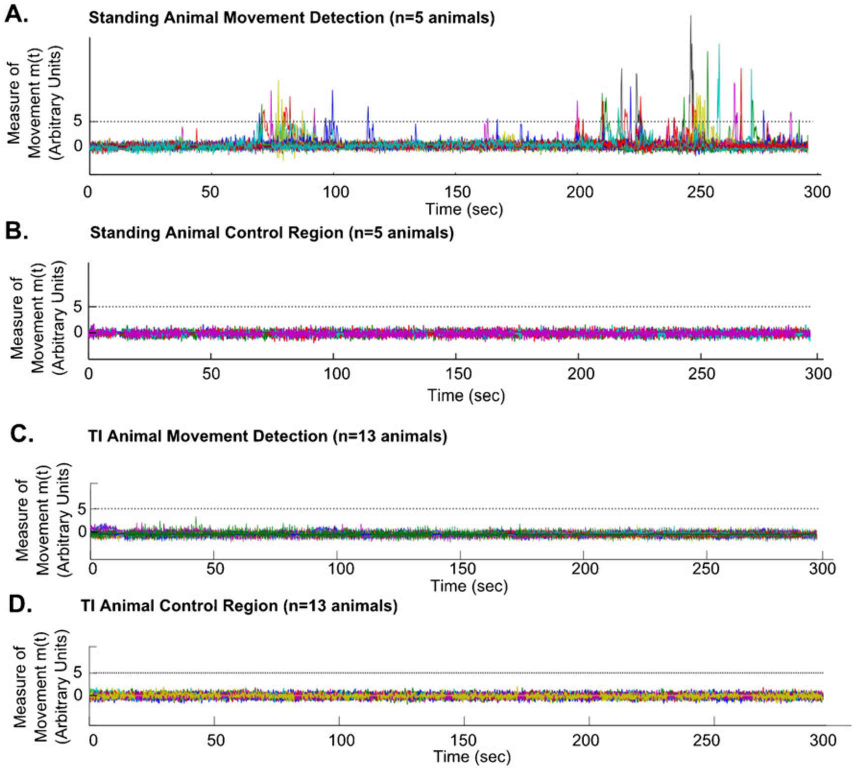

Fig. S1. (A) Detected movement in the standing, 'awake' experiments shows movements crossing the threshold set at 5 units (dotted line). (C) Detected movement in the TI animals shows no movement crossing the threshold. (B,D) Controls regions for the standing animals and TI animals respectively show no spuriously detected movements. Each color refers to the detrended, root mean square normalized movement time series *(m(t))* of one detection region; there were 5 detection regions per animal (left and right forlegs/hindlegs, palps).

Biology Open

Biology Open

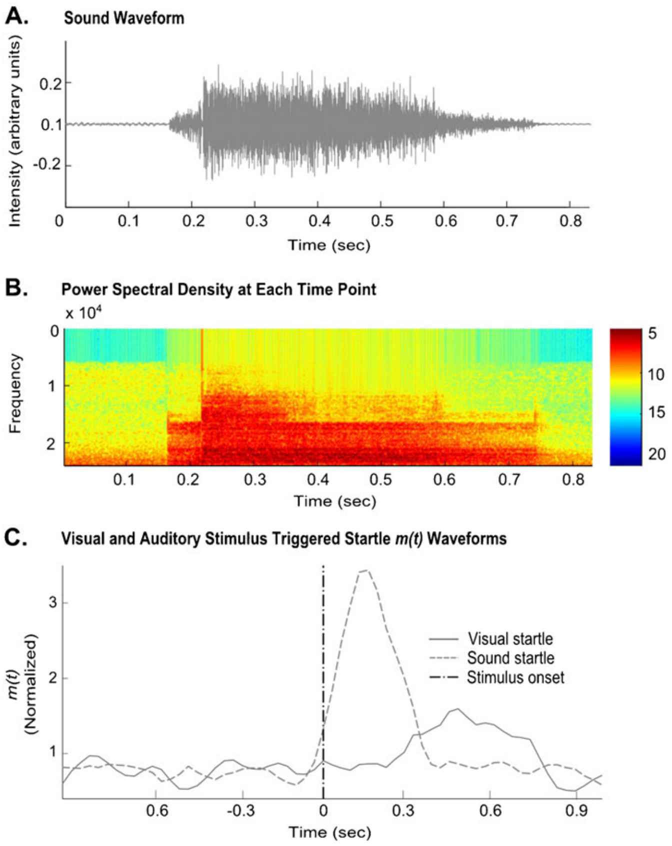

Fig. S2. (A) Waveform of the sound stimulus as recorded by the video-camera. (B) Spectrogram of the sound waveform computed using short-time Fourier transform. (C) The visual and auditory stimulus triggered startle *m(t)* waveforms show that the startle response to the sound stimulus is faster than the startle response to the visual stimulus.

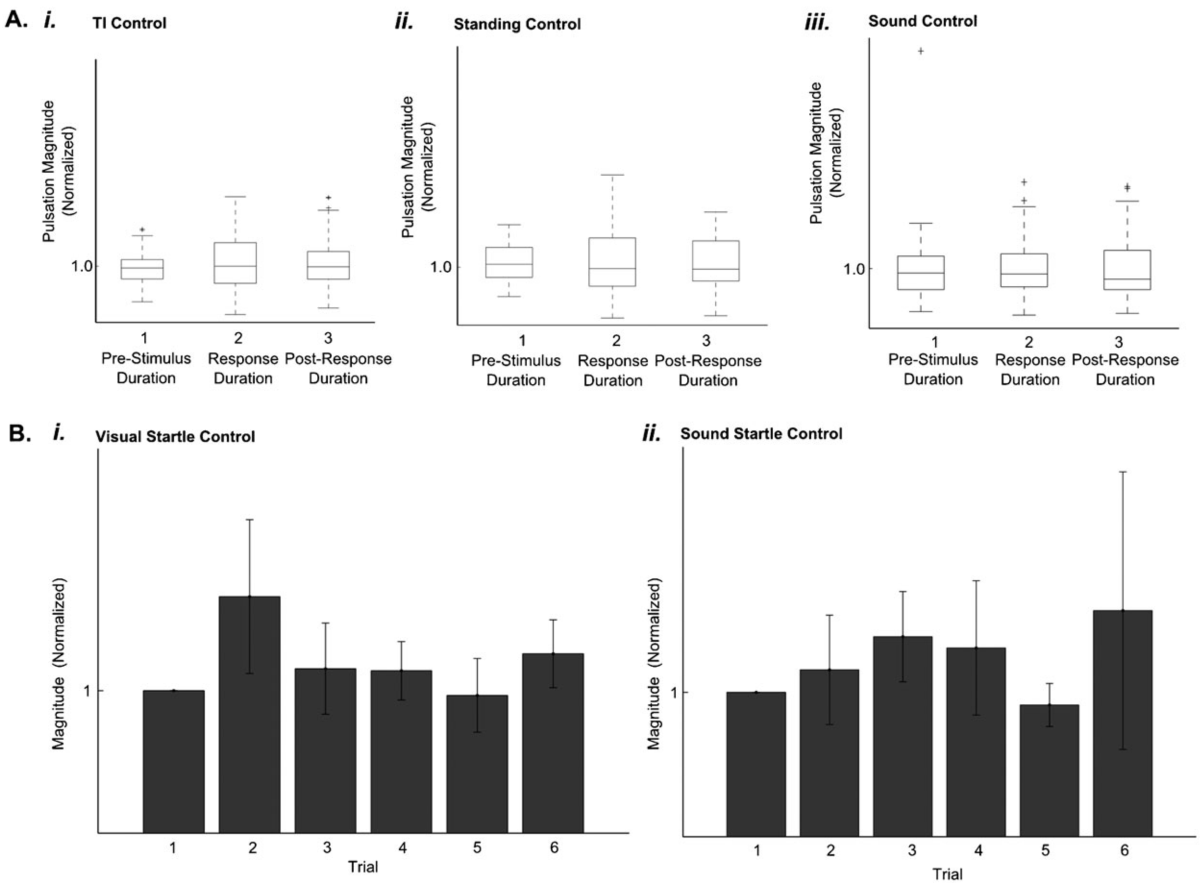

Fig. S3. (A) Control regions show no response to visual stimuli during TI (A*i*) and 'awake' (A*ii*) states, and no response to sound stimuli during TI (A*iii*) ($P>0.3$ by Kruskal-Wallis test of medians). (B) Control regions show no habituation in response to (B*i*) visual and (B*ii*) sound stimuli.

Biology Open

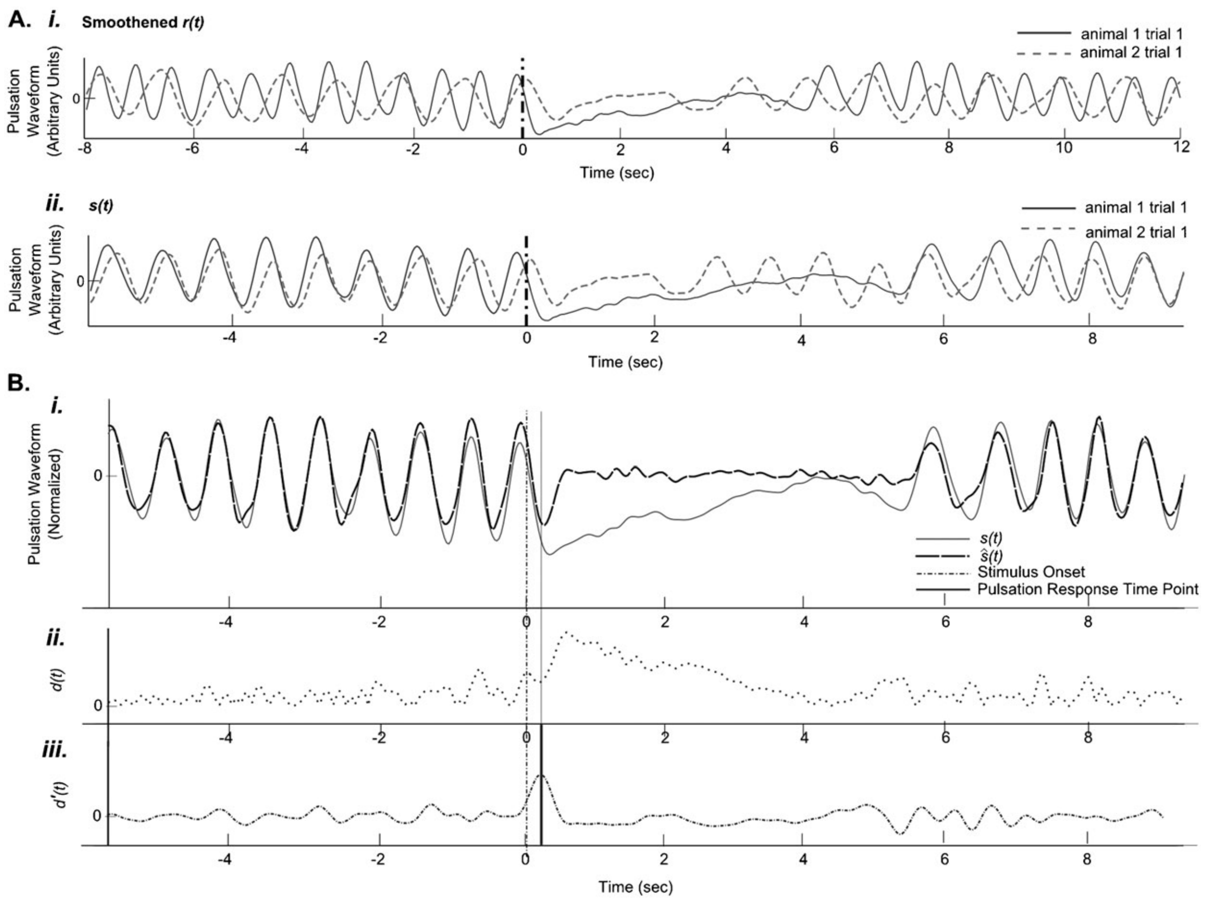

Fig. S4. (A*i*) Sample traces of smoothened stimulus triggered *r(t)* waveforms for two different animals. (A*ii*) Sample traces of frequency normalized stimulus triggered *r(t)* waveforms, labeled as *s(t)*, for the same animals as in (A*i*). (B) Procedure for locating the time point of onset of the response to the stimulus. First *s(t)* is differentiated, scaled and 90 degree phase shifted to get $\hat{s}(t)$. (B*i*,*ii*) $d(t)=|s(t)-\hat{s}(t)|$ is then calculated. (B*iii*) The time point after the stimulus onset where the differential of *d(t)* crosses a threshold is the onset of the pulsation response.

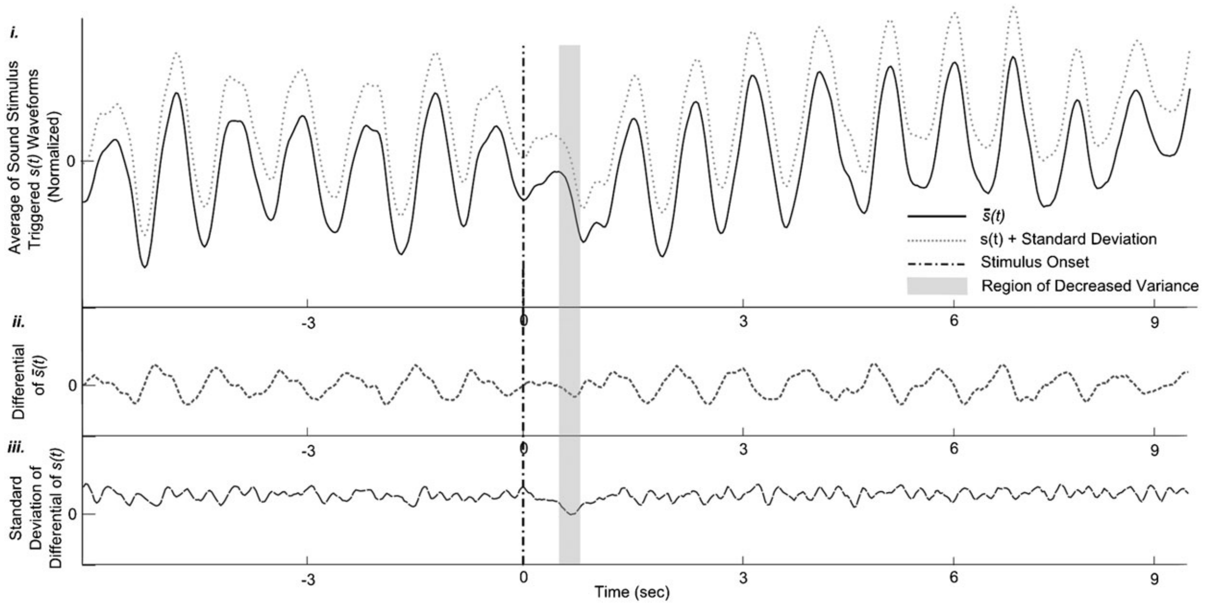

Fig. S5. (*i*) The average of sound stimulus triggered *s(t)* waveforms. (*ii*) The differential of *i* indicates a negative slope after the stimulus is given. (*iii*) A dip in the standard deviation of the differential indicates that this negative slope shows less variation over trials and animals (shaded band; $n=7$ animals, 5 trials per animal).

Biology Open

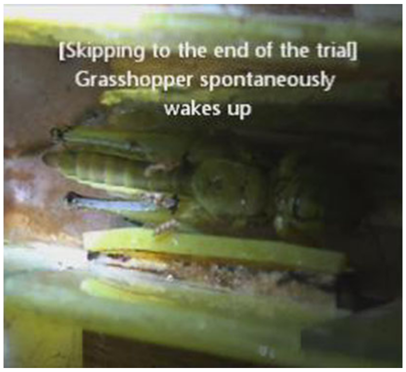

Movie 1. Response to translating visual stimuli in tonic immobile state.
